# Waterpipe smoke condensate activates autophagy in non-tumorigenic lung epithelial cells: Implications for inflammation and stemness

**DOI:** 10.1371/journal.pone.0358943

**Published:** 2026-09-24

**Authors:** Rania F. Zaarour, Bilal Azakir, Ayda S. Mahmood, Zohra Nizami, Husam Nawafleh, Yehya ElSayed, Salem Chouaib

**Affiliations:** 1 Thumbay Research Institute for Precision Medicine, Gulf Medical University, Ajman, United Arab Emirates; 2 Molecular and Translational Medicine Laboratory, Faculty of Medicine, Beirut Arab University, Beirut, Lebanon; 3 Department Chemistry, Wayne State University, Detroit, United States of America; 4 INSERM UMR 1356, Next-Generation Immuno-Oncology Research and Therapy in Pediatric and Adult Cancer, Gustave Roussy, Faculty of Medicine, University Paris-Saclay, Villejuif, France; "INSERM", FRANCE

## Abstract

Waterpipe smoking has emerged as a major global health concern, with incompletely characterized effects on bronchial epithelial cells. In this study, we investigated the cytotoxic and adaptive responses of non-tumorigenic human bronchial epithelial BEAS-2B cells exposed to waterpipe smoke condensate (WPSC). Increasing concentrations of WPSC reduced cell viability in a dose-dependent manner; however, exposure to 0.1% WPSC caused only a modest, transient reduction in viability that was no longer significant by 72 hrs and did not significantly induce apoptosis over 72 hrs, as determined by MTT (3-(4,5-dimethylthiazol-2-yl)-2,5-diphenyltetrazolium bromide) and Annexin V/Propidium Iodide (PI) apoptosis assays. To elucidate the adaptive mechanisms underlying this resistance, we examined autophagic flux and found that WPSC induced time-dependent accumulation of LC3-II and p62, which was enhanced by bafilomycin A1 cotreatment, indicating the activation of autophagy. This was accompanied by increased phosphorylation of AMPK and decreased phosphorylation of mTORC1, consistent with an autophagy-mediated stress response. Functionally, the inhibition of autophagy with bafilomycin A1 increased apoptosis and necrosis, suggesting that autophagy could play a protective role. Moreover, WPSC exposure induced DNA damage, as evidenced by increased γH2AX and 53BP1 nuclear foci, and elevated proinflammatory cytokine expression (IL-1β and CCL2). Finally, WPSC treatment upregulated the expression of the cancer stem cell-associated markers CD44 and CD133. Collectively, our findings demonstrate that subcytotoxic exposure to waterpipe smoke activates autophagy in non-tumorigenic lung epithelial cells. This adaptive mechanism may represent an early cellular event linking waterpipe smoke exposure to lung carcinogenesis.

## Introduction

Lung cancer is the most common cancer and the leading cause of cancer-related death worldwide [1], with tobacco smoking being its number one risk factor because of the large number of toxicants and carcinogens that it carries [2]. Despite the risks, several smoking modalities continue to evolve. Among these, waterpipe smoking (WPS) has long been used to attract users because of its social appeal and perceived lower harm. However, smoking waterpipes exposes users to more carbon monoxide and dramatically more smoke exposure than cigarettes do, in addition to the toxicants derived from burning charcoal [3].

Several studies have linked the effects of WPS to lung cancer through the activation of several mechanisms including, reactive oxygen species (ROS) generation, DNA damage, inflammation, cellular senescence, the expression of cancer stem cell (CSC) markers, and autophagy activation [4,5]). These effects collectively promote epithelial to mesenchymal transition (EMT) in cancer cells and impair cytotoxic immune responses [6–8]. While these effects parallel those induced by cigarette smoke [9], the early molecular and cellular changes that occur in normal lung cells following WPS exposure remain poorly understood. Defining these responses is important, as they may represent early adaptive events that predispose cells to malignant transformation.

By employing multiple regulatory mechanisms to protect against stress-induced damage, cells continuously monitor their environment to maintain homeostasis. Among these mechanisms is autophagy, a lysosome-mediated catabolic process. Autophagy can act as a suppressor or a driver of tumorigenesis; it can act as a tumor suppressor by removing damaged organelles and proteins but can also be exploited by cancer cells as a prosurvival mechanism under stress, including chemotherapy [10]. Autophagy and apoptosis often intersect to determine cell fate under stress. Apoptosis is a programmed cell death pathway that eliminates damaged cells. Apoptosis suppression is a hallmark of cancer, and a barrier to effective therapy [11].

Autophagy is tightly regulated by key signaling pathways, including AMP-activated protein kinase (AMPK) and mammalian target of rapamycin complex 1 (mTORC1). AMPK is activated when cellular ATP levels are low, and once activated, it suppresses the mTORC1 pathway, thereby promoting autophagy [12]. AMPK is further modulated by environmental stressors, including smoke exposure [13].

While studies have explored the effects of WPS in cancer cell lines, little is known about its impact on non-malignant bronchial cells, prior to the initiation of early tumorigenic events. In the present study, we investigated the effects of waterpipe smoke condensate (WPSC) on the non-tumorigenic human bronchial epithelial (BEAS-2B) cells to elucidate the early molecular events associated with WPS exposure. We focused on determining whether WPSC modulates autophagy, apoptosis, and the AMPK signaling pathway. Our findings demonstrate that low-dose WPSC activates an autophagic response associated with increased inflammation and cancer stem cell marker expression, while attenuating apoptosis. These results suggest that WPS exposure elicits adaptive mechanisms in non-tumorigenic lung epithelial cells that may promote survival under stress and contribute to early carcinogenic processes.

## Materials and methods

### Waterpipe smoke sampling

Waterpipe smoke collection was performed as previously described [6]. Briefly, the waterpipe head was loaded with 17.5 g of “mouassal” double apple flavored tobacco and sealed with a punctured aluminum foil. Heating the tobacco was achieved by placing two quick lighting charcoal briquettes on top of the foil. Smoke generation and collection were performed using an automated smoking robot (IREADY LLC) designed to replicate the human smoking behavior that simulates the human puffing process. Each puff lasted 5 seconds, followed by a 15-second interval between puffs, with the entire session comprising 80 puffs in total. The resulting smoke condensate was trapped on glass wool fibers that had been pre-conditioned and housed within a T-shaped collection tube.

### Cell line

BEAS-2B cells purchased in 2018 from (ECACC 95102433 RRID: CVCL_0168) were grown in BEGM media (cat. no. CC-3170; Lonza). Cells were regularly tested to ensure they are mycoplasma free. Cells were treated with 100 nM bafilomycin A1 (Cell Signaling, 54645, Danvers, MA, USA), where indicated.

### MTT assay

MTT was purchased from Abcam (MTT Assay Kit, Abcam ab211091, Cambridge, UK). BEAS-2B cells were seeded at a density ranging from 0.3–1 x 10^4^ cells/mL in 96 well plates. Cells were then treated with increasing concentrations of WPSC after 24 hrs of seeding for 24, 48 and 72 hrs. At the corresponding time of incubation, media was removed and replaced with 100 µL of the MTT solution and incubated for 4 hrs. Formazan crystals were solubilized in 100 µL of DMSO and absorbance was recorded using an ELISA reader (Thermo Fisher Scientific, Boston, MA, USA) at 570 nm. Cell proliferation rates were calculated by comparing with the control cells.

### RNA extraction/cDNA and qPCR

RNA was extracted and purified using Easy Blue (cat. no. 17061; Intron Biotechnology, Inc.) following the instructions of the manufacturers. Nanodrop and 1% agarose gel electrophoresis were used to determine the RNA concentration and quality. cDNA synthesis was conducted with high-capacity cDNA reverse transcription kit (cat. no. 4374966; Thermo Fisher Scientific Inc.). qPCR was conducted using SYBR-Green (cat. no. 4309155; Thermo Fisher Scientific Inc.) using AB 7500 FAST Real-Time PCR system. ∆∆Cq method was used to analyze the data [14]. Forward (F) and reverse (R) primers are as follows: ACTIN forward (5’-TCCTTCCTGGGCATGGAGT-3’) and ACTIN reverse (5’-AGCACTGTGTTGGCGTACAG-3’); CCL2 forward, (5′-CAGCCAGATGCAATCAATGCC-3′) and CCL2 reverse, (5′-TGGAATCCTGAACCCACTTCT-3′), IL1B forward, (5′-TTCGACACATGGGATAACGAGG-3′) and IL1B reverse, (5′-TTTTTGCTGTGAGTCCCGGAG-3′); CD44 forward, (5′-TGCCGCTTTGCAGGTGTATT-3′) and CD44 reverse, (5′-CCGATGCTCAGAGCTTTCTCC-3′); CD133 forward, (5′- AGTCGGAAACTGGCAGATAGC-3’) and CD133 reverse (5’-GGTAGTGTTGTACTGGGCCAAT-3’); IL-6 forward, (5′-ACTCACCTCTTCAGAACGAATTG-3′) and IL-6 reverse, (5′-CCATCTTTGGAAGGTTCAGGTTG-3′).

### Immunoblotting

Cells were plated on 10 cm culture dishes. For proteins extraction cells were first rinsed with 1X ice-cold PBS and then lysed in 100 µl of RIPA (150 mM NaCl, 0.1% TX-100, 0.5% NaDOC, 0.1% SDS, 50 mM Tris-CL pH 8.0) in presence of protease inhibitor cocktail (cat. no. P2714; Sigma-Aldrich; Merck KGaA). Proteins were quantified using Pierce BCA protein assay kit (cat. no. 23225; Thermo Fisher Scientific Inc.). 8–12 µg of proteins were separated on 10% or 12% SDS-PAGE and then transferred to a nitrocellulose membrane (cat. no. GE10600004; Sigma-Aldrich; Merck KGaA) depending on the target protein molecular weight. Membranes were blocked with 5% BSA in TBST (10 mM Tris, pH 8.0, 150 mM NaCl, 0.5% Tween-20) for 60 min and then incubated with appropriate antibody overnight at 4°C according to their manufacturer’s sheets. Densitometric analysis of the Western blot bands was performed using ImageJ software (Version 1.54, National Institutes of Health, USA).

### Antibodies used in this study

Mouse monoclonal anti-human phospho-histone H2AX (cat. no. 05–636; 1:1,000 dilution; Merck Millipore), rabbit anti-human histone H2AX (cat. no.ab11175; 1:2,000 dilution; abcam), rabbit anti-human 53BP1 (cat. no. 4937; 1:1,000 dilution; Cell Signaling Technology), mouse monoclonal anti-gizzard β-actin (cat. no. sc-47778; 1:5,000 dilution; Santa Cruz Biotechnology), mouse monoclonal anti-human p62/SQSTM1 (cat. No. 88588S, 1:1,000 dilution; Cell Signaling Technology), rabbit anti-human LC3 (cat. No. 4108S, 1:1,000 dilution; Cell Signaling Technology), rabbit anti-human AMPK (cat.no. 2532S, 1:1,000 dilution; Cell Signaling Technology), rabbit anti-human phospho-AMPK (cat.no. 2535S; Cell Signaling Technology), rabbit anti-human mTOR (cat.no. 2972S; Cell Signaling Technology) or rabbit anti-human phospho-mTOR (cat.no. 5536S Cell Signaling Technology) and DAPI (cat. no. D1306; 1:36,000 dilution; Thermo Fisher Scientific Inc.). Secondary antibodies used were goat anti-mouse Alexa Fluor 568 (A11004; 1:1,000 dilution; Thermo Fisher Scientific, Inc.), goat anti-rabbit Alexa Fluor 488 (cat. no. A11034; 1:1,000 dilution; Thermo Fisher Scientific Inc.), goat anti-mouse Alexa Fluor 488 (cat. no. A11001; 1:1,000 dilution; Thermo Fisher Scientific Inc.), goat anti-rabbit Alexa Fluor 568 (cat. no. A11011; 1:1.000 dilution; Thermo Fisher Scientific Inc.), Anti-mouse IgG HRP-linked antibody (cat.no.7076 Cell Signaling; 1:7000), and Anti-rabbit IgG HRP-Linked Antibody (Cat.no.7074 Cell Signaling; 1:7000.).

### Immunofluorescence

Cells were fixed in 4% paraformaldehyde (cat. no. 28906; Thermo Fisher Scientific Inc.) in 1X PBS at room temperature for 10 min. Following 1X PBS wash, cells were permeabilized with 0.1% TX-100 in PBS for 15 min. Then, cells were blocked in 2% BSA in 1X PBS for 1 hr at room temperature before staining with primary and secondary antibodies as described below. After washing, cells were mounted on glass slides using Prolong gold antifade reagent (cat. no. P36930; Thermo Fisher Scientific, Inc.) and visualized on Zeiss LSM 800 with Airyscan.

### Flow cytometry

Apoptosis was assessed using the APC Annexin V Apoptosis Detection Kit with Propidium Iodide (PI) (Biolegend, 640914 San Diego, CA, USA). Cells were seeded at a density of 100,000 cells per 35 mm dish (Eppendorf 0030 700.112, Hamburg, Germany). Following WPSC treatment, the cells were collected at the indicated timepoints by trypsinization and subsequently washed with 1 × PBS prior to labeling with Annexin V-APC and PI following the manufacturer’s protocol. Acquisitions of 10,000 cells was performed using a Bio-Rad S3E Cell Sorter and data were processed using the FCS Express flow cytometry program (De Novo Software, Pasadena, CA, USA). Annexin V-positive cells were classified as apoptotic.

### Statistical analysis

Statistical analyses were carried out using GraphPad Prism Software version 9.3.1 (GraphPad Software, Inc, San Diego, CA, USA). Data are expressed as means ± SEM. Comparisons involving two factors (e.g., concentration and time) were analyzed by two-way ANOVA, whereas single-factor were analyzed by one-way ANOVA. In all cases, ANOVA was followed by Tukey’s post hoc test for multiple comparisons. P ≤ 0.05 was considered significant.

### Use of artificial intelligence tools

ChatGPT with GPT-4o (OpenAI) was used to generate the schematic illustration presented in Fig 6 and for language editing support. The authors reviewed, edited, and verified the scientific accuracy of all AI-assisted content and take full responsibility for the final manuscript.

## Results

### 0.1% WPSC does not induce apoptosis in BEAS-2B cells

**Fig 1:**
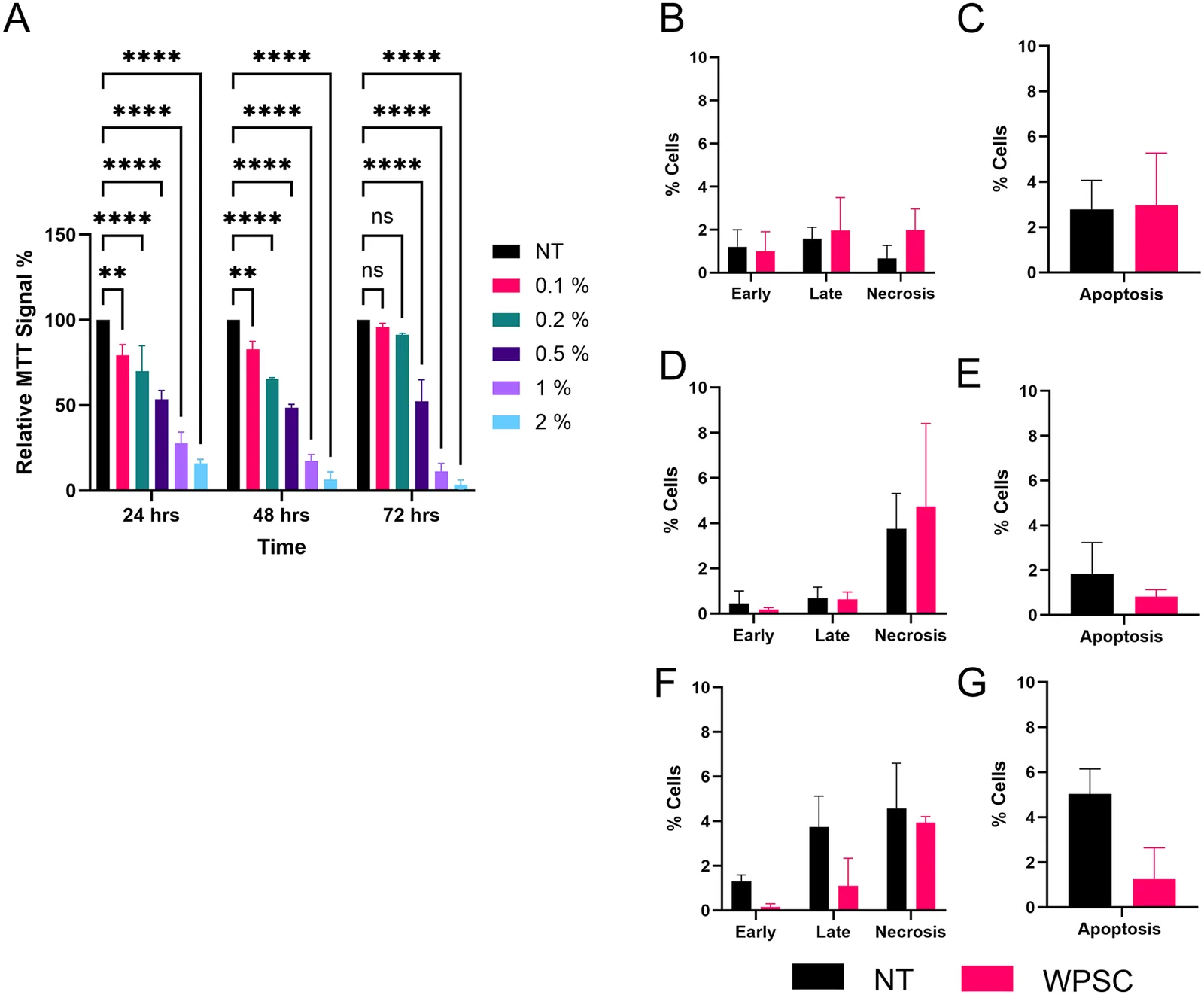
WPSC results in cell death in a dose-dependent manner. (A) MTT assay following exposure to increasing concentrations of WPSC at 24-, 48-, and 72- hrs. (B-G) Annexin/PI analysis of the effects of 0.1% WPSC following 24 hrs (B, C), 48 hrs (D, E), and 72 hrs (F, G). Panels B, D and F display the proportions of early apoptotic (Annexin positive), late apoptotic (double positive) and necrotic (PI positive) cells. Panels C, E and G display total apoptosis (early and late combined) under each condition. Results represent means of three independent experiments and data represent mean ± standard error of mean. ns: non-significant, *P ≤ 0.05, **P ≤ 0.01, ***P ≤ 0.001 and ****P ≤ 0.0001.

We tested the cytotoxic effects of increasing concentrations of waterpipe smoke condensate (WPSC) on BEAS-2B cells at 24, 48, and 72 hrs. WPSC reduced MTT absorbance in a dose dependent manner. At both 0.1% and 0.2% WPSC a modest but significant reduction in MTT absorbance was observed at 24 and 48 hrs, but not at 72 hrs (Fig 1A). Annexin V/PI staining revealed that 0.1% WPSC did not induce significant increase in early apoptosis, late apoptosis, or necrosis at any time point examined (Figs 1B–G). 0.1% was selected for all subsequent experiments as the lowest concentration of interest, thereby minimizing non-specific cellular stress.

### Waterpipe smoke exposure results in activation of autophagic flux in BEAS-2B cells

Because 0.1% WPSC did not induce apoptosis, we hypothesized that an adaptive mechanism, such as autophagy, might be activated in response to WPSC exposure. We therefore analyzed autophagic flux by monitoring LC3-II protein accumulation in the presence of WPSC and bafilomycin A1, a late-stage autophagy inhibitor that blocks autophagosome-lysosome fusion, thereby preventing lysosomal degradation of autophagosome contents.

**Fig 2:**
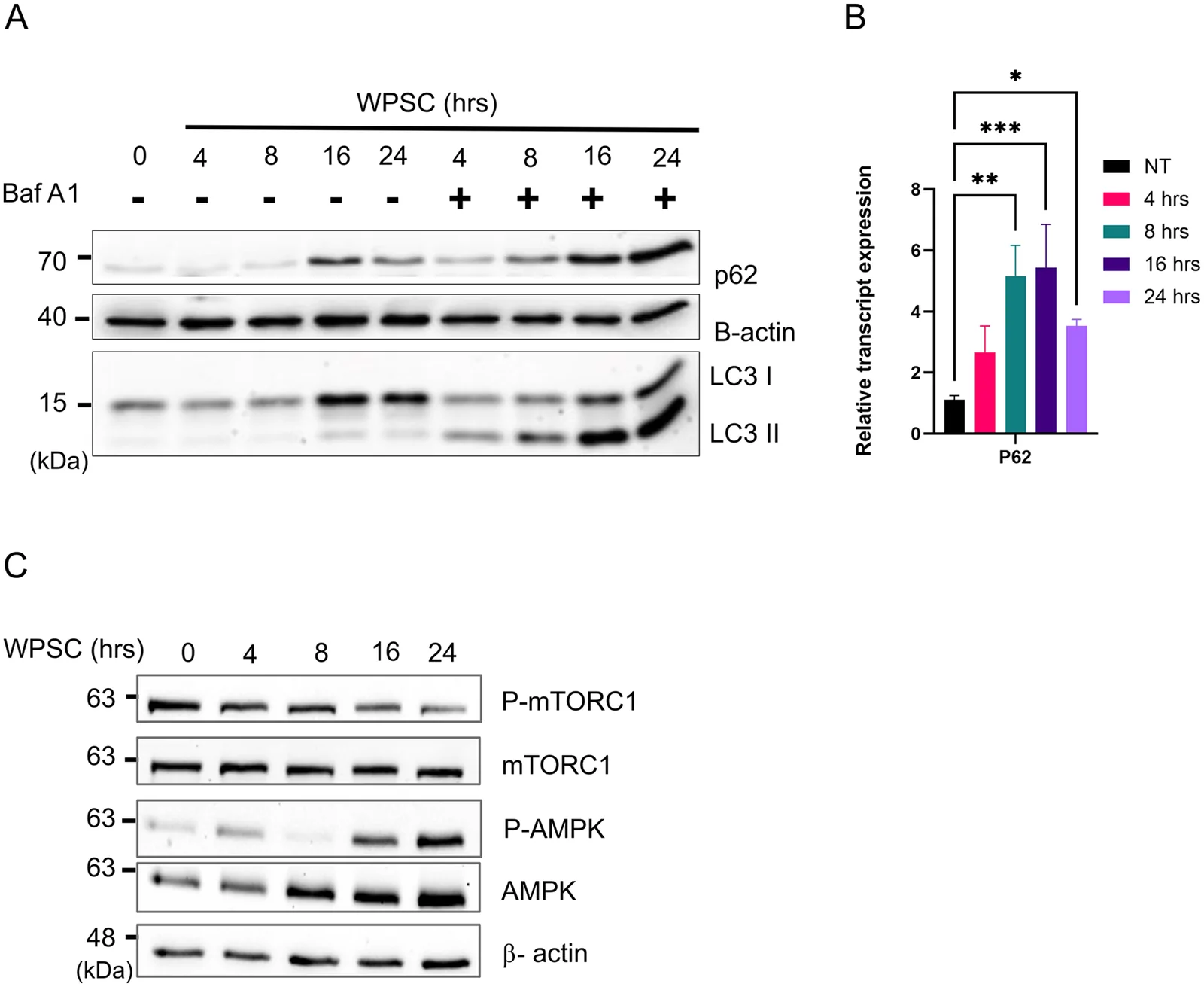
WPSC increases autophagic flux. BEAS-2B cells were treated with 0.1% WPSC for 4, 8, 16 and 24 hrs. (A) Protein expression levels of LC3 and p62 were determined with or without 100 nM bafilomycin A1, (B) gene expression levels of p62 were determined by qPCR*.* (C) Protein levels of phospho-AMPK, total AMPK, phospho-mTORC1 and total m-TORC1 were assessed by western blot. β-actin was used as a loading control for the western blots. Results represent means of three independent experiments and data represent mean ± standard error of mean. *P ≤ 0.05, **P ≤ 0.01 and ***P ≤ 0.001.

WPSC treatment induced a time-dependent accumulation of LC3-II levels at 8 hrs which peaked at 24 hrs. This effect was further enhanced with bafilomycin A1 cotreatment, indicating that autophagy was activated (Fig 2A). Similarly, p62 protein and transcript levels were elevated post WPSC treatment, p62 accumulated further upon bafilomycin A1 co-treatment (Fig 2A), indicating that p62 continues to be delivered to and degraded by autophagy under WPSC exposure. The concurrent increase in p62 transcript indicates that the net rise in basal p62 protein reflects transcriptional upregulation rather than impaired clearance (Figs 2 A and B). Additionally, we observed an increase in phospho-AMPK and a decrease in phospho-mTORC1 following 16 hrs and 24 hrs of WPSC exposure which is consistent with autophagy activation (Fig 2C). Quantification of the western blots is presented in S1 Fig.

### Inhibiting autophagy results in increased BEAS-2B cell death

**Fig 3:**
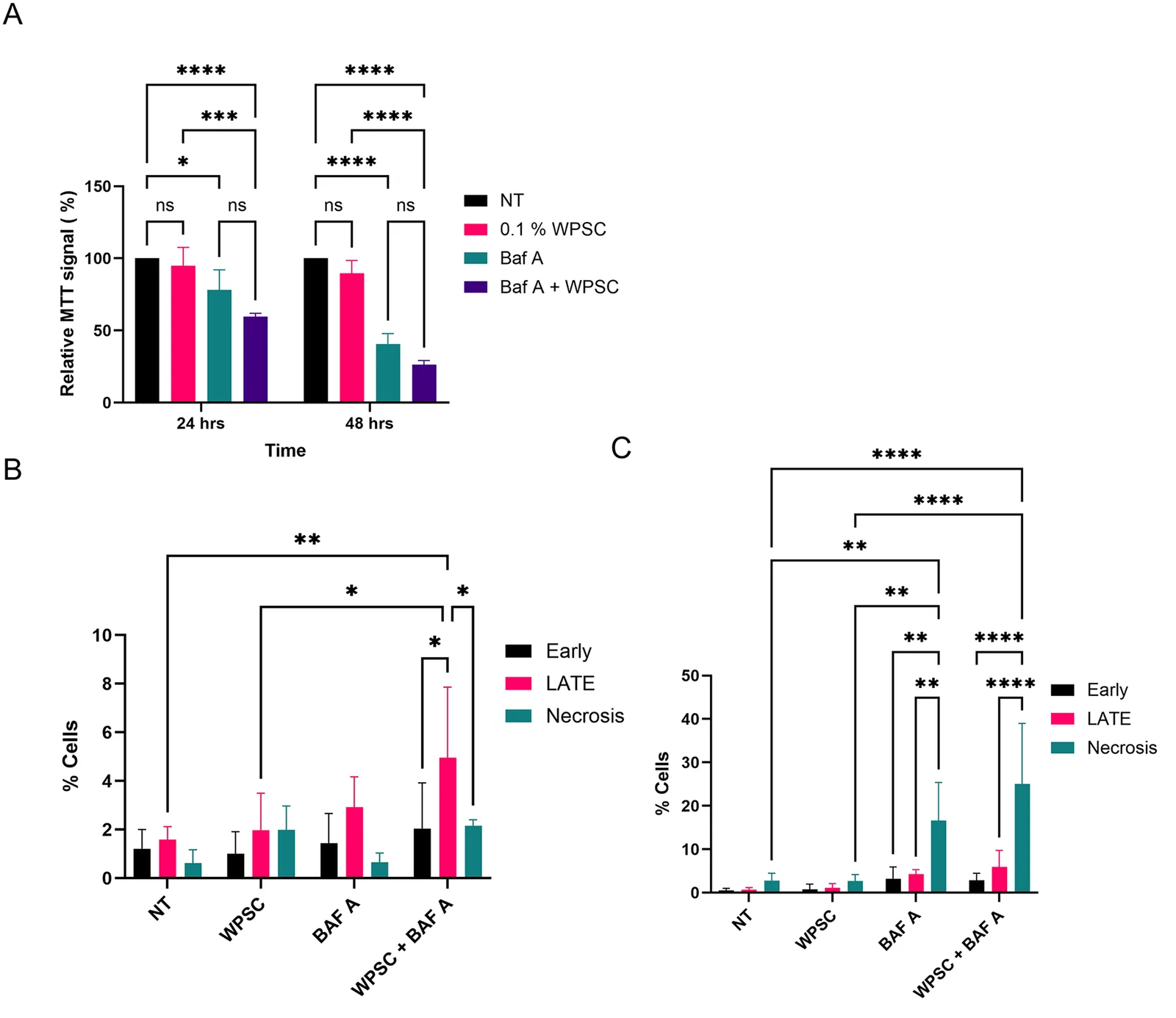
Inhibiting autophagy increases cell death. BEAS-2B cells were treated with 100 nM bafilomycin A1 with or without 0.1% WPSC for a duration of 24 hrs or 48 hrs. Cell viability was assessed by MTT assay (A). Apoptosis was evaluated by FACS analysis of Annexin / PI-positive cells at 24 hrs (B) or 48 hrs (C). Percentages of cells in each population are displayed. Results represent means of three independent experiments and data represent mean ± standard error of mean. *P ≤ 0.05, **P ≤ 0.01, ***P ≤ 0.001 and ****P ≤ 0.0001.

Autophagy activation may influence a cell’s susceptibility to apoptosis. To determine whether WPSC-induced autophagy promotes cytoprotection, we analyzed whether blocking autophagy alters apoptotic outcomes. BEAS-2B cells were treated with WPSC in the presence of bafilomycin A1 for 24 hrs and 48 hrs. While WPSC did not induce cell death, cotreatment with bafilomycin A1 markedly induced cell death (Fig 3A). Furthermore, BEAS-2B cells treated with WPSC in the presence of bafilomycin A1 for 24 hrs presented a significant increase in late apoptotic cells compared with those treated with WPSC alone (Fig 3B). At 48 hrs treatment we observed a significantly greater increase in the dead cell population (Necrotic cells, PI+) (Fig 3C). However, this increase was not significant relative to bafilomycin A1 alone, as bafilomycin A1 by itself already induced substantial cell death (Figs 3A–C)

### WPSC increases DNA damage and inflammatory marker levels in BEAS-2B cells

**Fig 4:**
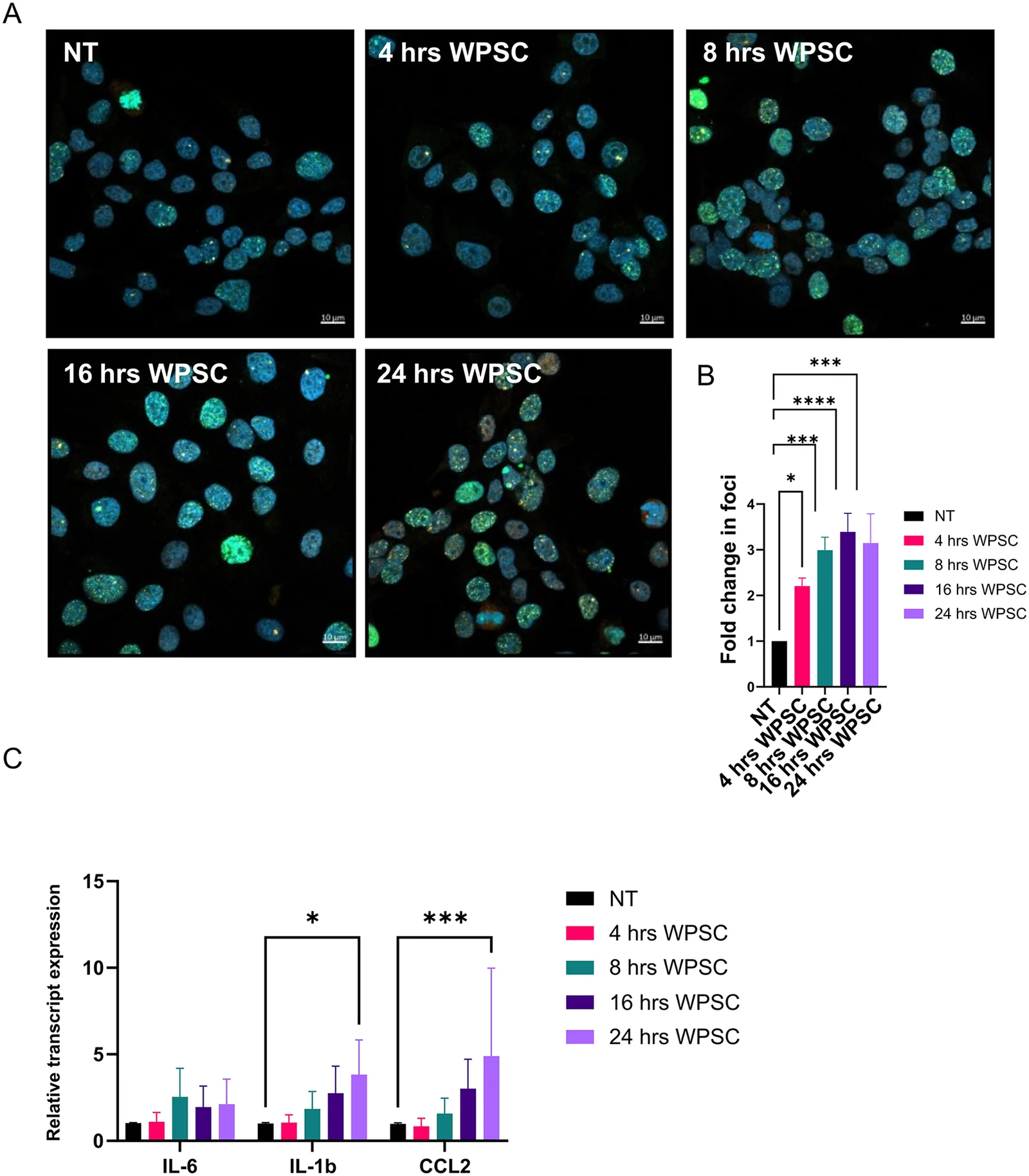
WPSC induces inflammation and DNA damage. BEAS-2B cells were treated with 0.1% WPSC, for the indicated time points. DNA damage was measured via analysis of the foci formation for γH2AX (green) and 53 BP1 (orange) in WPSC-treated cells; representative images are shown (A). Quantification of the fold change in foci number, scored in 100 cells, is shown in B. Scale bar = 10 µm. Gene expression was measured by qPCR for the inflammatory markers IL-6, IL-1β and CCL2. Results represent means of three independent experiments and data represent mean ± standard error of mean. *P ≤ 0.05, **P ≤ 0.01,***P ≤ 0.001, and ****P ≤ 0.0001.

DNA damage can induce autophagy and inflammatory signaling, which are associated with pro-survival mechanisms [15]. DNA damage was measured by quantifying 53 BP1/γH2AX foci formation. We observed an increase in the number of nuclear foci for both γH2AX and 53 BP1 in BEAS-2B cells treated with WPSC (Figs 4A and B), reflecting the accumulation of DNA damage.

DNA damage and inflammation are closely linked to tumorigenesis. We therefore measured key inflammatory markers in BEAS-2B cells exposed to WPSC. IL-6, IL-1β and CCL2 were selected as inflammatory markers given their well-documented roles in smoke-induced lung inflammation and their association with lung cancer progression [16]. IL-1β has been directly linked to increased lung cancer risk through persistent inflammatory signaling [17], while CCL2 has been specifically reported to be elevated following waterpipe smoke exposure [18]. In addition, we previously demonstrated that WPSC exposure in lung cancer cell lines increased the expression of inflammatory genes including IL-6, IL-1β, and CCL2 [6]. We observed a significant increase in the proinflammatory cytokines IL-1β and CCL2 but not IL-6 (Fig 4C).

### Waterpipe smoke induces the expression of stemness-associated markers in BEAS-2Bcells

**Fig 5:**
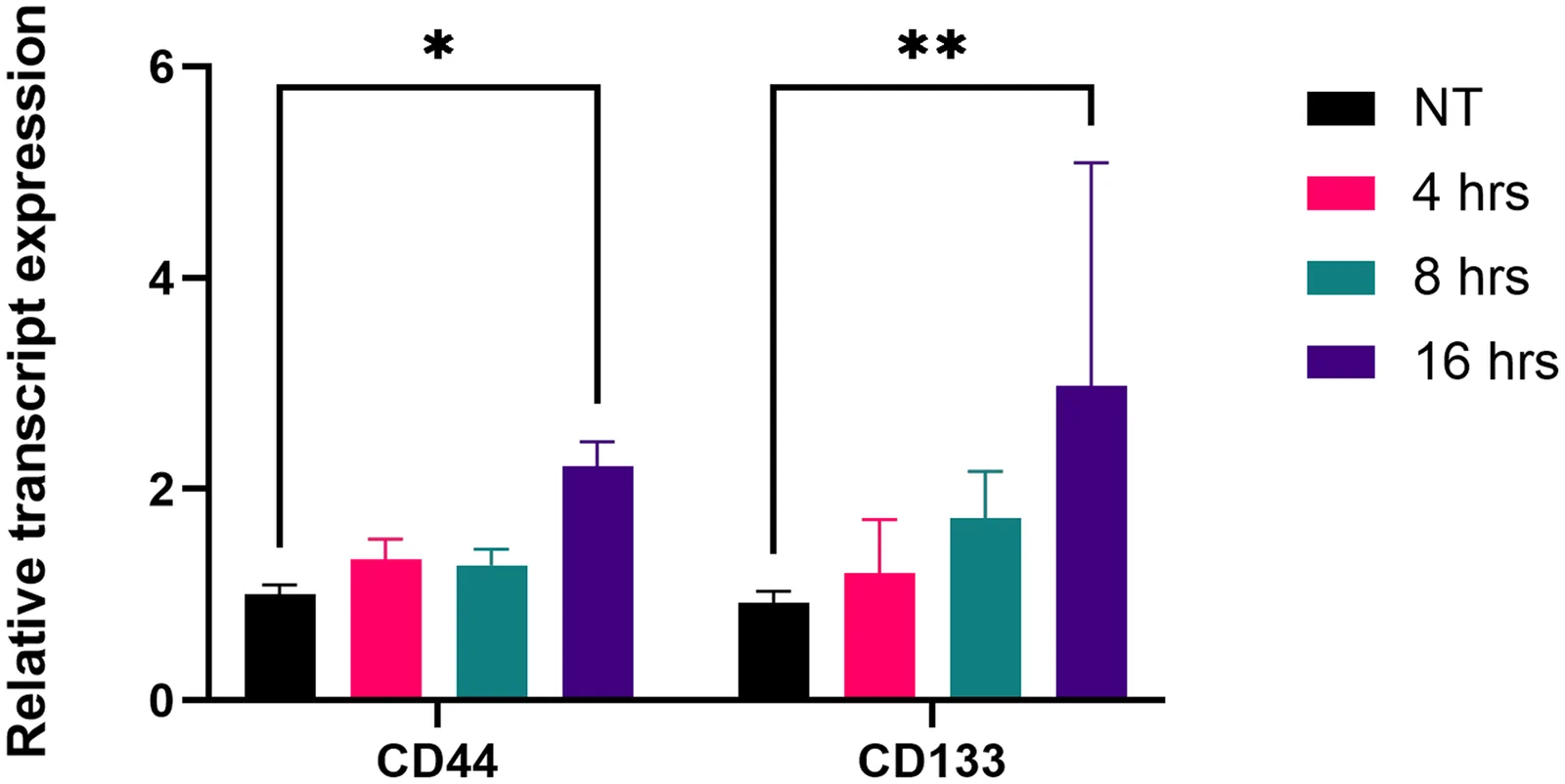
WPSC increases the expression of CSC markers. BEAS-2B cells were treated with 0.1% WPSC at the indicated time points. Gene expression was measured by qPCR for the CSC markers CD44 and CD133. Results represent means of three independent experiments and data represent mean ± standard error of mean. *P ≤ 0.05, **P ≤ 0.01 and ***P ≤ 0.001.

Pathways involved in inflammation can promote the emergence of CSC populations. CD44 and CD133 were selected as representative CSC markers based on their well-documented roles in lung cancer stemness, tumor-initiating capacity, and resistance to therapy [19]. Our results demonstrate that the CSC markers CD44 and CD133 were significantly increased following 16 hrs of WPSC treatment (Fig 5).

## Discussion

**Fig 6:**
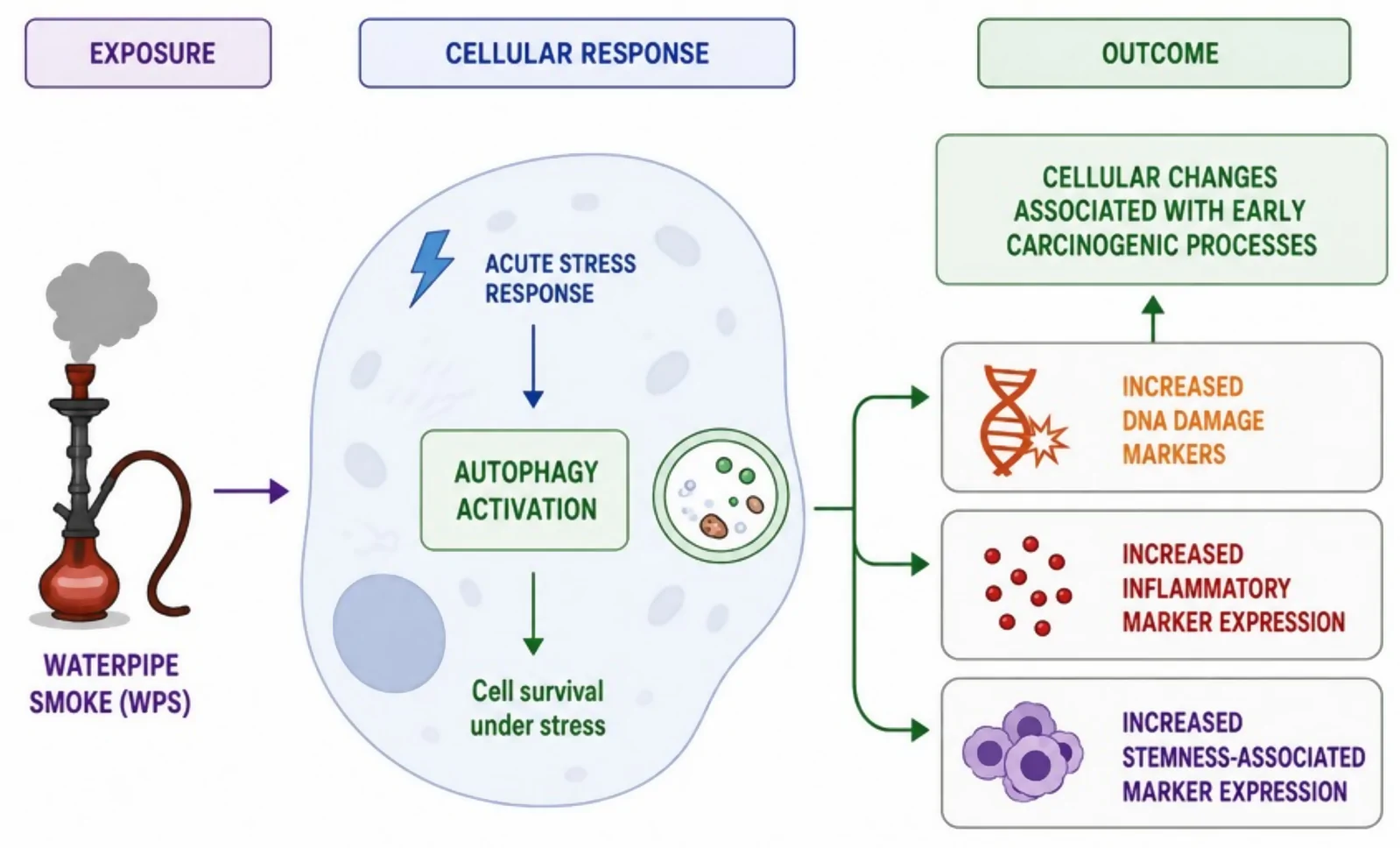
Graphical summary of the cellular responses to WPSC in BEAS-2B cells. Subcytotoxic WPSC exposure induces an acute stress response associated with autophagy activation and cell survival under stress, together with increased DNA damage markers, inflammatory marker expression, and stemness-associated marker expression. These findings suggest that WPSC may promote a pro-tumorigenic cellular state. The schematic is intended to summarize associated responses observed in this study and does not indicate proven causality or oncogenic transformation.

In this study we demonstrate that WPSC activates autophagy in non-tumorigenic BEAS-2B lung epithelial cells, accompanied by DNA damage, inflammatory signaling and upregulation of stemness-associated markers expression (Fig 6). These findings are consistent with and extend our group’s previous work demonstrating that WPSC activates autophagy in lung cancer cell lines [4] and now provide evidence that this response also occurs in non-tumorigenic cells. Despite differences in the chemical composition of each smoking modalities, cigarette smoke as well increases inflammatory markers and activates autophagy in BEAS-2B cells [20,21] through ROS-mediated AMPK activation and mTOR suppression in lung cells [22], suggesting a shared upstream mechanism between cigarette and waterpipe smoke. Consequently, cancer that develops from WPSC and cigarette smoke exposure may exhibit overlapping molecular signatures, a research area that merits further investigation.

In the context of lung disease, autophagy may either confer a cytoprotective role or drive pathological pathways [23], though chronic smoke exposure from WPS impairs lysosomal acidification [24,25], suggesting that the autophagy we observe may represent an early adaptive response before such dysfunction sets in.

Our study was conducted using an *in vitro* model with short duration treatment, of up to 72 hrs. Other work has demonstrated that long-term cigarette smoke exposure in mice results in the activation of AMPK and downstream autophagy [26]. Together, these results suggest that AMPK is a regulator of smoke-induced stress adaptation.

A limitation of the present study is the use of the BEAS-2B cell line as a model of non-tumorigenic lung bronchial epithelium. Although widely used in respiratory research, Han et al. [27] and Lee and Ryu [28] demonstrated that BEAS-2B cells share characteristics with mesenchymal stem cells, including surface marker profiles and differentiation potential, raising questions about their purely epithelial identity. Therefore, results obtained using this model should be interpreted cautiously, ideally validated in primary bronchial epithelial cells. Additionally, genetic inhibition of autophagy via ATG5 or ATG7 silencing and long-term functional assays such as colony formation and migration/invasion would be needed to further substantiate the association between autophagy and the observed changes in DNA damage, inflammatory cytokines, or stemness markers. Our findings should therefore be interpreted as suggestive of an early adaptive role of autophagy in WPSC-exposed non-tumorigenic lung cells.

Our study highlights potential mechanistic links between smoking and lung cancer initiation. Lung cancer is characterized by rapid growth and metastasis with limited treatment strategies. Its high tumor mutational burden contributes to intratumoral heterogeneity [29], which poses a major challenge for effective treatment and fosters therapy resistance. Understanding the early effects of WPS in lung cells can support public health policy makers in strengthening the smoking cessation regulations, which is the most effective strategy to reduce the burden of smoking on the population health outcome.

## Supporting information

S1 Fig Quantification of western blots. Western blot band intensities were quantified by densitometric analysis using ImageJ. (A) p62 protein levels were normalized to β-actin and quantified from at least three independent experiments at 4, 8, 16, and 24 hrs. (B) LC3-II protein levels were normalized to β-actin and quantified from two independent experiments; therefore, no statistical analysis was performed. (C) Phospho-mTOR and phospho-AMPK levels were quantified as [p-AMPK/β-actin]/[AMPK/β-actin] and [p-mTORC1/β-actin]/[mTORC1/β-actin], from two independent experiments at 16 and 24 hrs. Therefore, no statistical analysis was performed. Data are presented as mean ± SEM. *P ≤ 0.05, **P ≤ 0.01, and ***P ≤ 0.001.
(TIFF)

S2 Fig Raw_images. (TIFF)
